# Activation of the hypoxia pathway in breast cancer tissue and patient survival are inversely associated with tumor ascorbate levels

**DOI:** 10.1186/s12885-019-5503-x

**Published:** 2019-04-03

**Authors:** Elizabeth J. Campbell, Gabi U. Dachs, Helen R. Morrin, Valerie C. Davey, Bridget A. Robinson, Margreet C. M. Vissers

**Affiliations:** 1 Mackenzie Cancer Research Group, Department of Pathology and Biomedical Science, University of Otago, Christchurch, 8011 New Zealand; 2 Centre for Free Radical Research, Department of Pathology and Biomedical Science, University of Otago, Christchurch, 8140 New Zealand; 3 Cancer Society Tissue Bank, University of Otago, Christchurch, 8011 New Zealand; 4 Christchurch Breast Cancer Patient Register, Christchurch Hospital, Christchurch, 8011 New Zealand; 5 Canterbury Regional Cancer and Haematology Service, Canterbury District Health Board, Christchurch, and Department of Medicine, University of Otago, Christchurch, 8011 New Zealand

**Keywords:** Hypoxia-inducible factor-1, Ascorbate, Breast cancer, Disease-free survival

## Abstract

**Background:**

The transcription factor hypoxia inducible factor (HIF) -1 drives tumor growth and metastasis and is associated with poor prognosis in breast cancer. Ascorbate can moderate HIF-1 activity in vitro and is associated with HIF pathway activation in a number of cancer types, but whether tissue ascorbate levels influence the HIF pathway in breast cancer is unknown. In this study we investigated the association between tumor ascorbate levels and HIF-1 activation and patient survival in human breast cancer.

**Methods:**

In a retrospective analysis of human breast cancer tissue, we analysed primary tumor and adjacent uninvolved tissue from 52 women with invasive ductal carcinoma. We measured HIF-1α, HIF-1 gene targets CAIX, BNIP-3 and VEGF, and ascorbate content. Patient clinical outcomes were evaluated against these parameters.

**Results:**

HIF-1 pathway proteins were upregulated in tumor tissue and increased HIF-1 activation was associated with higher tumor grade and stage, with increased vascular invasion and necrosis, and with decreased disease-free and disease-specific survival. Grade 1 tumors had higher ascorbate levels than did grade 2 or 3 tumors. Higher ascorbate levels were associated with less tumor necrosis, with lower HIF-1 pathway activity and with increased disease-free and disease-specific survival.

**Conclusions:**

Our findings indicate that there is a direct correlation between intracellular ascorbate levels, activation of the HIF-1 pathway and patient survival in breast cancer. This is consistent with the known capacity of ascorbate to stimulate the activity of the regulatory HIF hydroxylases and suggests that optimisation of tumor ascorbate could have clinical benefit via modulation of the hypoxic response.

## Background

The transcription factor, hypoxia-inducible factor (HIF)-1, is the master regulator for tumor adaptation to hypoxia generated by rapid cell growth and chaotic and fragile blood vessel networks [1–3]. HIF-1 upregulates many genes controlling glycolysis, intracellular pH (e.g. carbonic anhydrase IX [CA-IX]), angiogenesis (via vascular endothelial growth factor [VEGF]) and cell life and death pathways (e.g. Bcl-2/adenovirus E1B 19 kDa interacting protein 3 [BNIP-3]) [1–3]. Markers of HIF-1 activation are associated with increased tumor growth, resistance to chemotherapy and radiation, increased metastasis and poor prognosis, including in breast cancer. [2, 4–13]. Numerous studies have indicated a distinct survival advantage associated with reduced expression of HIF-1α, the regulatory subunit of HIF-1, in patients with breast cancer [5–9, 11–16].

HIF-1 activity is controlled by post-translational hydroxylation of HIF-1α; proline hydroxylation at P402 and P564 targets the protein for proteasomal degradation and hydroxylation of asparagine N803 prevents the formation of an active transcription complex [17–19]. The hydroxylases responsible for this regulation are Fe-containing 2-oxoglutarate-dependent dioxygenases (2-OGDDs) [20–22] that require 2-oxoglutarate and oxygen as substrates and Fe and ascorbate as cofactors. HIF-1 is activated when hydroxylation of HIF-1α is compromised [22–24].

Ascorbate levels influence HIF-1 activation [23, 25–27]. In cell culture, increased intracellular ascorbate decreases the extent of HIF-1 activation under conditions of mild-moderate hypoxia and metabolic disturbance similar to those seen in tumors [25, 28–31]. In ascorbate-dependent Gulo^−/−^ knock-out mice, increased ascorbate availability reduced tumor growth, dampened HIF-1 activity, decreased areas of hypoxia and necrosis, and normalised tumor vasculature [29, 30, 32]. Retrospective analysis of human tumor tissue samples has revealed that increased ascorbate levels were associated with reduced HIF-1 pathway activity in endometrial [27], colorectal [26], papillary renal cell carcinoma [33] and thyroid tumor tissue [34], and with improved disease-free survival in colorectal cancer patients [26]. This data suggests that increased ascorbate availability dampens the tumor HIF-1 response and may mitigate the effects of tumor hypoxia. However, whether a similar relationship exists in breast cancer is unknown.

Breast cancer is the leading cause of cancer mortality for women [35]. HIF-1 is a major driver of breast cancer development and metastasis, and there is great interest in the potential to mitigate tumor hypoxia in this disease [2, 4–13]. Given previous findings, referred to above, that increasing cellular vitamin C content can moderate the hypoxic response, it is of interest to determine whether tumor ascorbate can influence the HIF-1 response in breast cancer.

There is some evidence from epidemiological studies that suggests that ascorbate intake could impact on breast cancer outcomes. Patient survival advantage with respect to ascorbate has been observed in a number of prospective observational studies that have monitored dietary vitamin C intake [36–42]. These observations differ from other studies that suggest that multivitamin use (that includes vitamin C) is associated with increased risk of developing breast cancer [43, 44]. However, determining the effect of a single component in a multivitamin analysis is subject to error and it is unclear whether the effect observed in these studies is associated with vitamin C. The most recent meta-analysis concluded that increased vitamin C intake is likely to be associated with a reduced risk of total and breast cancer-specific mortality [45]. However, none of these studies have measured ascorbate levels in either patient plasma or tumors, and there is no proposed mechanism of action to explain how increased ascorbate could impact on breast cancer progression and outcomes.

To determine whether tumor ascorbate levels vary in breast cancer and whether levels are associated with HIF-1 activation, we have undertaken a retrospective analysis of breast cancer tissues to quantify the ascorbate content in tumor and adjacent uninvolved breast tissue harvested from breast cancer patients at surgery. Ascorbate levels were related to the activation of the HIF-1 pathway, to patient survival, to tumor pathology, and the expression of the sodium dependent vitamin C transporter, SVCT-2. We hypothesise that tissue ascorbate levels are associated with HIF-1 pathway activity in breast cancer, and that low tumor ascorbate levels may have clinical implications.

## Methods

### Materials

MDA-MB-231 human breast cancer cells were from American Type Culture Collection, Cryosite Distribution, Australia. Anti- human HIF-1α, CA-IX, BNIP-3, and β-actin were from R&D Systems, Minneapolis, USA; anti-human SVCT-2 was from Atlas Antibodies, Bromma, Sweden; anti-goat or anti-mouse HRP-conjugated antibodies were from DAKO, Australia. Pre-cast gels, buffers and solutions were from Invitrogen, Carlsbad, CA, USA, and all other chemicals from Sigma-Aldrich, St Louis, MO, USA. The VEGF DuoSet ELISA kit was from R&D Systems, Minneapolis, USA.

### Ethical approval

Ethical approval was granted by the University of Otago Human Ethics Committee, New Zealand (H15/093). Tissue samples were collected and approved for use by the Cancer Society Tissue Bank, Christchurch (CSTB) (Ethical Approval 16STH92 and P.15105). All donors gave written informed consent for sample collection, storage and use, along with access to their related clinical information. In addition, all experiments were performed in accordance to relevant guidelines and regulations. Ethnicity was self-reported, allowing multiple ethnic affiliations. A prioritized ethnic classification system was used and women were classified as Māori/Pacific if this was declared in any of their affiliations, otherwise ethnicity was classified as non-Māori/non-Pacific.

### Patient selection

Tumor samples were included from patients who had undergone breast cancer surgery for invasive ductal carcinoma (IDC) at Christchurch hospital, New Zealand and become CSTB donors between 2002 and 2014. The patient cohort is an unselected group and there was no dietary or supplement intervention in this group. IDC tumors and paired normal tissue samples from 52 patients were selected to cover a range of grades and stages in both pre- and post-menopausal women. Tissue was taken from the same breast, with normal tissue taken at a distance of about 10 cm from the tumor. Most tumors contained both IDC and non-invasive ductal carcinoma in situ (DCIS). As per CSTB standard operating procedures, banked tissues were dissected and snap frozen in liquid nitrogen within an hour of surgical removal. The demographic and clinicopathological details (age, stage, grade, receptor status, nodal status, vascular invasion, necrosis) were from diagnostic pathology reports. Necrosis was only reported for the DCIS component of the tumor, as is standard.

### Follow-up and survival analysis

Women with breast cancer were managed at Christchurch hospital according to nationally accepted guidelines. Patients at risk of local recurrence were referred for radiation, and those at risk of distant spread were referred for systemic adjuvant therapies (chemotherapy based on anthracyclines and taxanes). Women with receptor positive tumors were referred for appropriate treatment (endocrine treatment, trastuzumab), following international guidelines.

Patient follow up data was collated from the Breast Cancer Registry. Disease-free survival was calculated with the date of reported metastases or local recurrence as endpoint. Disease-specific mortality was calculated from the date of primary surgery to the date of death, with cause of death recorded as breast cancer.

### Sample preparation for ascorbate, cellular DNA and VEGF measurements

Frozen tissue samples were homogenised into a fine powder in liquid nitrogen using a pre-cooled mortar and pestle, transferred into pre-weighed tubes and wet weight measured. Tumor homogenate (10–100 mg) was resuspended in 200 μL of phosphate buffer and used for ascorbate (160 μL aliquot), and DNA and VEGF analyses (40 μL aliquot).

### Ascorbate analysis

Tissue ascorbate was measured using reverse-phase HPLC with electrochemical detection (Dionex Ultimate HPLC, with Dionex detector) [26, 27, 29]. Briefly, ascorbate in the tissue homogenate was stabilised by the addition of 1:1 volume of 0.54 M perchloric acid containing 50 mM diethylene triamine penta-acetic acid and the protein precipitate removed by centrifugation at 10,000 g for 10 min. A standard curve of 1.25–40 μM ascorbate was analysed with each run.

### Cellular DNA and VEGF analysis

DNA content of the homogenate was measured with 1 mg/ml propidium iodide and fluorescence measurement at 544–590 nm relative to a standard curve of purified calf thymus DNA [26]. Human VEGF was measured in the same homogenate using a DuoSet ELISA kit.

### Sample preparation for immunoblot analysis

Due to the high fat content of the breast tissue, an optimised protein extraction procedure was developed. Homogenised tissue (10–100 mg) was immediately immersed in 200 μL of ice cold RIPA buffer (150 mM NaCl, 1.0% IGEPAL® CA-630, 0.5% sodium deoxycholate, 0.1% SDS, 50 mM Tris, pH 8.0 and containing complete mini protease inhibitors) on ice. Samples were further sheared by passing 5 times through a 21G needle and sonication for ~ 30 s. The homogenate was centrifuged at 10,000 g for 10 min at 4 °C, the lipid layer removed with an insulin needle, the remaining sample was further sonicated and centrifuged at 10,000 g for 10 min at 4 °C. The protein present in the final supernatant was precipitated with 9 volumes of ice-cold acetone on ice, collected by centrifugation at 2000 g for 10 min and resuspended in fresh RIPA buffer. The protein concentration was measured using the Direct Detect® Spectrometer (Merck-Millipore, Germany).

### Immunoblot analysis

Tumor and matched normal samples were standardized to 40 μg of protein/ well, using 4x Bolt Laemmli buffer, containing dithiothreitol. Samples were separated by electrophoresis on a 4–12% BisTris gradient SDS gel and transferred to polyvinylidene fluoride membrane. To allow normalization of the signal between Western blots, the same positive control (whole cell lysate harvested from MDA-MB-231 cells exposed to 1% O_2_) was loaded onto each blot. Membranes were probed with antibodies against human HIF-1α (1/800 dilution), CA-IX (1/800 dilution), BNIP-3 (1/1000) and β-actin (1/10000 dilution), and with secondary anti-goat or anti-mouse HRP-conjugated antibodies (1/10000). Blots were visualized with enhanced chemi-luminescence (ECL) reagent and bands quantified using Alliance software.

### HIF-1 pathway score

Relative protein expression was obtained by normalising band density with respect to β-actin, and to a positive control (MDA-MB-231 cells exposed to 1% O_2_) to control for exposure between blots. To obtain a HIF-1 pathway score for each tumor, the relative expression of each protein was determined as percent of the maximum expression, and the individual scores for HIF-1α, CA-IX, BNIP3 and VEGF were combined (HIF-1 pathway score = HIF-1α + CA9 + BNIP3 + VEGF). This approach provided a signature for the overall transcriptional activity of HIF-1 in each tumor.

### Statistical analysis

Data were analysed with SPSS and GraphPad Prism with the alpha level set at 0.05. The Schapiro-Wilks normality test was used to determine the distribution of each variable. Normal vs tumor levels were compared using non-parametric Wilcoxon matched pairs signed rank. Associations between ascorbate and clinicopathological variables, or the HIF pathway, were assessed using Mann-Whitney or unpaired t-tests, for non-parametric and parametric data, respectively. Kaplan-Meier survival curves were analysed using Log rank (Mantel-Cox) test. Spearman’s correlations were used to probe relationships between clinicopathological variables and ascorbate or the HIF pathway, and between tumor ascorbate and SVCT-2.

## Results

### Description of patient cohort

Tumors and adjacent normal breast tissue from women with primary infiltrating ductal carcinoma were included for analysis. Tumors were collected for tissue banking as part of routine treatment for breast cancer, and the patient group is unselected for any variations in fruit and vegetable or supplement intake. Most patients declared themselves as non-Māori/non-Pacific (79% European, 6% other or undeclared) with 15% Māori or Pacific, reflecting the ethnic makeup of the Canterbury region, New Zealand (stats.govt.nz). The median age of the cohort was 60 years, with 50% under 55 years and presumed to be pre-menopausal.

### Tissue banking procedures and storage

The banking procedure is designed to minimise loss of tissue markers and all samples were snap frozen within an hour of surgery. We monitored for effect of handling and storage, and noted that the HIF-1α protein content of both tumor and normal tissues did not vary over time (*R*^2^ = 0.0038 and 0.0152, respectively) which ranged from 2 to 14 years at -80 °C. We have previously demonstrated that ascorbate is stable in the tumor tissue during the banking procedure, and that there is no loss of tissue ascorbate upon extended storage at -80 °C [27].

### Hypoxia pathway in breast cancer tumors

**Fig. 1:**
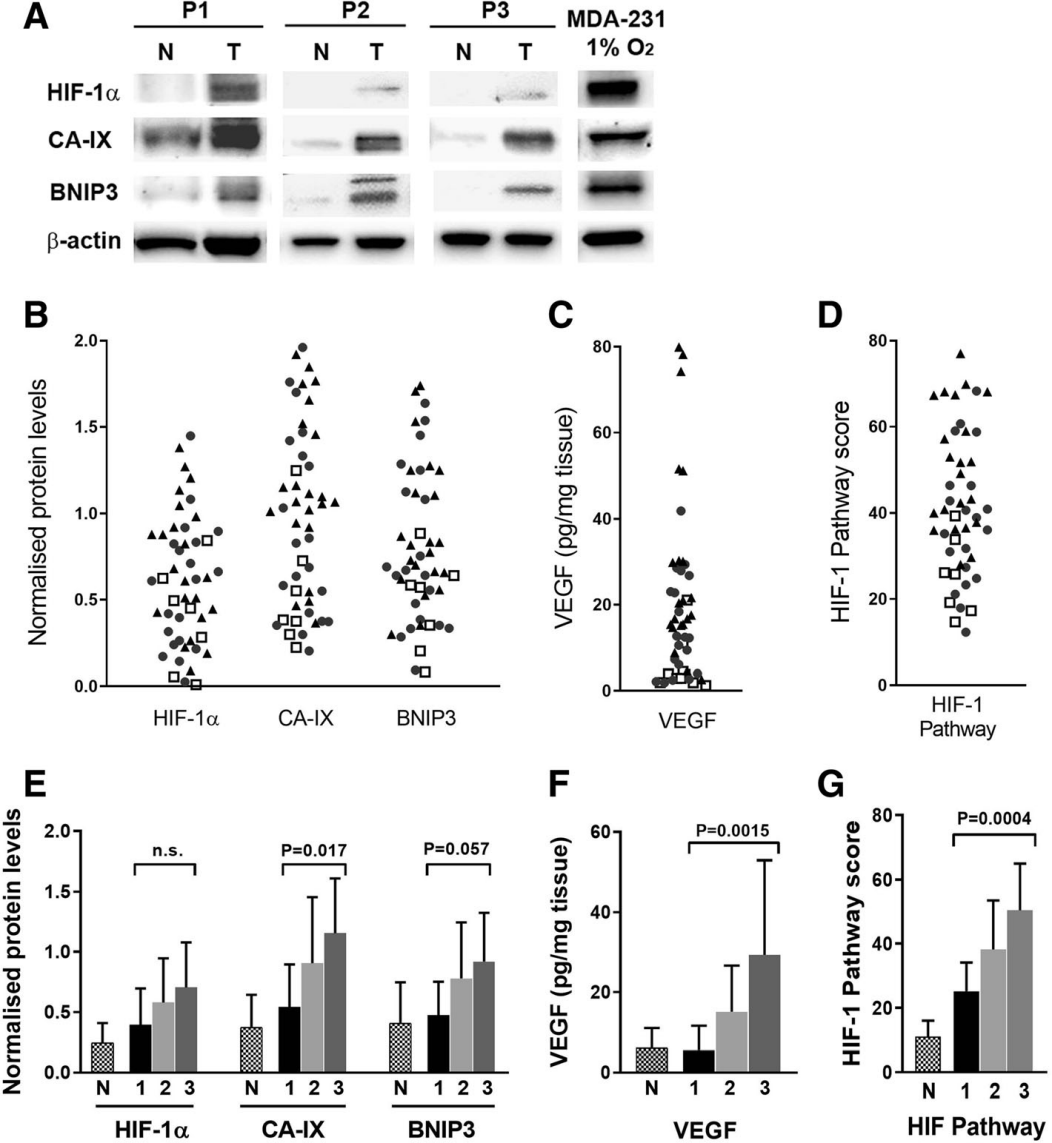
Detection of HIF-1α and HIF-dependent proteins in human breast tumor tissue. VEGF was measured by ELISA and HIF-1α, CA-IX and BNIP3 were analysed in tumor and matching normal breast tissue by western blotting (**a**). For quantification of the proteins on western blots, band density was measured with ImageJ. Levels of individual proteins were normalised to the internal loading control, β-actin. A positive control (MDA-MB-231 cells exposed to 1% O_2_) was loaded onto each gel to compare between blots and used to normalise development of protein bands between gels. The data in (**a**) are shown to exemplify the variable expression of HIF-1 pathway proteins in three individual patients (P1-P3) in tumor (T) and normal (N) tissue. Quantification was carried out with bands below saturation levels. **b**, **c** Relative expression of HIF-1α and downstream proteins for individual tumors are shown. Grade 1 (□) (*n* = 9), Grade 2 (●) (*n* = 20), Grade 3 (▲) (*n* = 22) tumors are indicated. The HIF Pathway score (**d**) for each tumor sample was derived by combining the individual scores for each protein from B and C, as follows: HIF-1 pathway score = HIF-1α + CA9 + BNIP3 + VEGF. **e**, **f**, **g** HIF-1 dependent proteins and HIF-1 Pathway score by tumor grade, showing means ± S.D., and uninvolved tissue in comparison. Significant difference across the three grades was assessed by ANOVA

HIF-1α protein was detected in 94% (49/52) of tumor samples, and levels were significantly increased compared with the matched normal tissue sample (*p* < 0.001, paired t-test). Similarly, CA-IX, VEGF and BNIP3 levels were elevated in tumor compared to normal tissue, (*p* < 0.001, *p* < 0.001, *p* < 0.001 respectively, paired t-test) (Fig. 1a, e and f). Expression levels of each protein in the tumor samples covered a broad range, as illustrated in Fig. 1a, b, c and we noted a significant increase with tumor grade for each protein (Fig. 1e, f).

Use of the HIF-1 pathway score indicated associations with markers of tumor aggression, including grade (*p* < 0.005), stage (*p* = 0.042), vascular invasion (*p* = 0.023) and DCIS necrosis (*p* = 0.001), as well as with invasive tumor size (Table 1). When individual scores for HIF-1α or individual transcriptional marker proteins were considered, these associations were not significant (Fig. 1 and Table 1). Breast cancer subtypes could affect the expression of the HIF pathway proteins, as high levels of CA-IX expression have been reported in basal-like and triple negative breast cancers [46, 47]. We noted increased levels of HIF-1α in hormone receptor negative tumors (*p* = 0.02, Table 1), and CA-IX levels were also significantly higher in HR^−^, compared to HR^+^ tumors (relative protein levels of 0.84 ± 0.48 and 1.44 ± 0.38, *P* = 0.001, *n* = 39 and *n* = 10, respectively). There was no difference in the range of expression of BNIP3 or VEGF between HR^+^ and HR^−^ tumors. These results combined for significantly higher HIF-1 pathway scores for the HR^−^ tumors (Table 1).

**Table 1:** Relationship of tumor HIF-1α protein levels and HIF-1 pathway score with clinicopathological parameters

Parameter		n	HIF-1α protein^a^	*p* value	HIF-1 pathway score^a,b^	*p* value
Grade	1	7	0.39 ± 0.30	0.14	25.2 ± 9.0	**0.004**
	2	20	0.58 ± 0.37		38.2 ± 15.3	
	3	22	0.70 ± 0.37		50.5 ± 14.5	
Tumor stage	1	16	0.59 ± 0.45	0.70	37.9 ± 16.7	**0.042**
	2	31	0.60 ± 0.33		42.2 ± 15.6	
	3	2	0.83 ± 0.31		68.7 ± 1.8	
Node	0	17	0.53 ± 0.33	0.29	38.9 ± 14.7	0.37
	1	32	0.65 ± 0.39		43.4 ± 17.5	
Vascular invasion	0	22	0.55 ± 0.43	0.28	36.5 ± 16.2	**0.023**
	1	26	0.67 ± 0.32		47.2 ± 15.3	
	na	1				
DCIS Necrosis	0	17	0.49 ± 0.31	0.14	31.5 ± 11.1	**0.001**
	1	27	0.65 ± 0.35		47.0 ± 15.8	
	na	5				
Tumor size^c^	Pearson correlation	49	*R* = 0.101	0.49	*R* = 0.348	**0.014**
Hormone Receptor Status^d^	HR^+^	39	0.55 ± 0.34	**0.02**	38.1 ± 15.3	**0.001**
	HR^−^	10	0.85 ± 0.40		56.6 ± 13.0	

^a^Mean ± SD from *n* = 49 patients, *p* values from ANOVA. Significance is indicated when *p* <0.05

^b^HIF-1 pathway score calculated from relative protein levels of HIF-1α, CA-IX, BNIP-3 and VEGF

na, not available

^c^Tumor size, invasive component only, excluding DCIS

^d^Hormone receptor HR^+^, ER+(PR+/−, Her2+/−) and includes both Luminal A and B; HR^−^, ER-PR-(Her2+/−) and includes Her2-enriched and basal-like tumours

### Impact of HIF-1 activation on patient survival

**Fig. 2:**
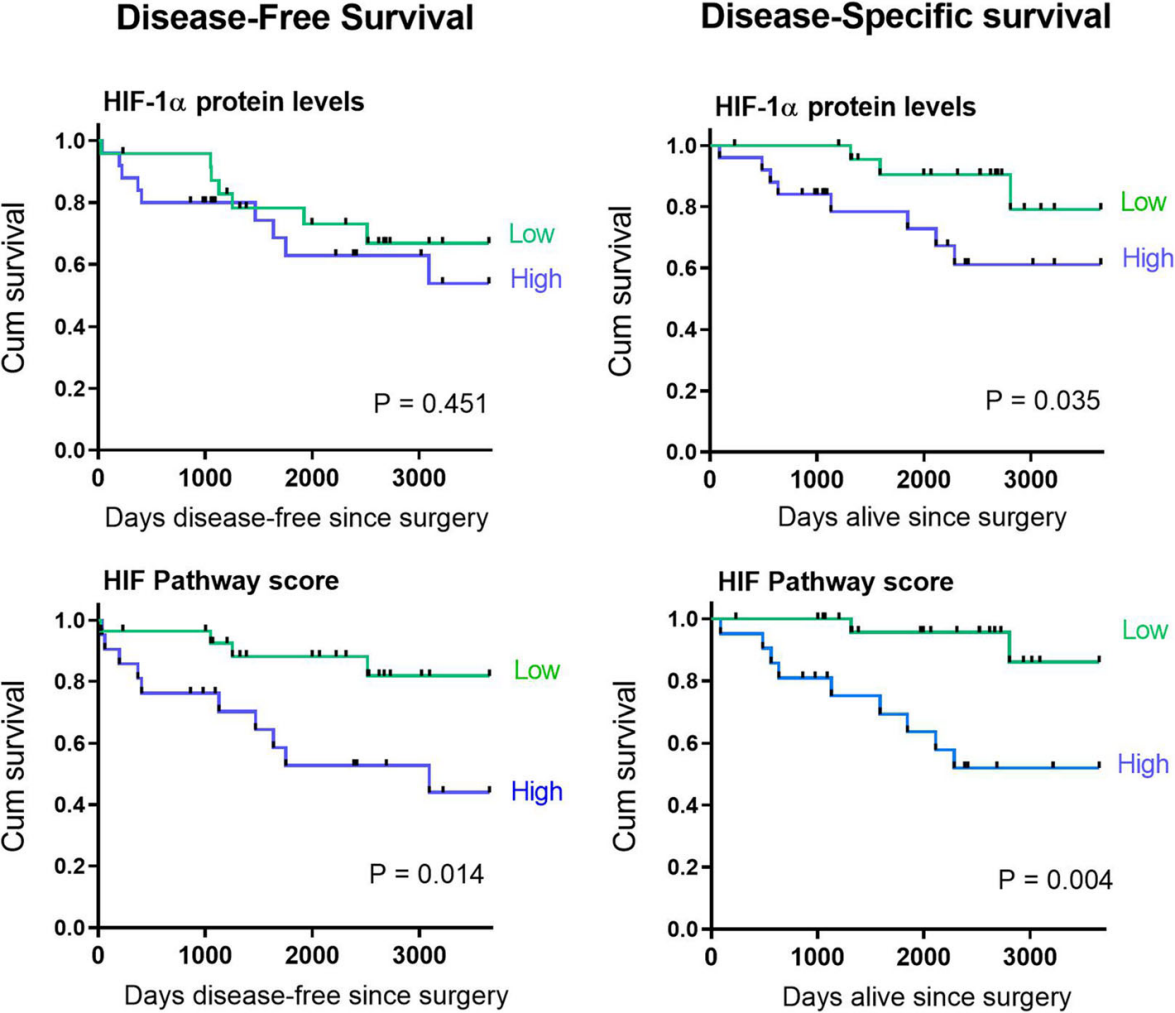
Patient disease-free and disease-specific 10 year survival associated with HIF-1α protein expression or HIF-Pathway score. Kaplan-Meier plots with above and below the mean levels of HIF-1α or the mean of the HIF Pathway score. Patient numbers in each group: high HIF-1α, *n* = 25; low HIF-1α, *n* = 24; low HIF Pathway, *n* = 28; high HIF Pathway, *n* = 21. Significant difference was determined by log-rank (Mantel Cox) analysis

The patient cohort was followed up for a median of 2400 days following surgery to record incidence of local recurrence or metastasis, or death from breast cancer. During follow-up, 14 patients recorded metastases (mainly to bone and lung), one recorded a local recurrence, and 13 patients died from breast cancer. Tumor HIF-1α protein levels were associated with 10-year disease-specific survival (*P* = 0.035) but not with disease-free survival (*P* = 0.451) (Fig. 2). In comparison, increased HIF Pathway scores were associated with a marked reduction for both disease-free (*P* = 0.014) and disease-specific patient survival (*P* = 0.004, Fig. 2).

### Ascorbate levels in tumor and normal tissue and relationship with clinicopathological features

Tissue ascorbate levels were related to DNA content to standardise for the cellular content of tumor or normal tissue. This was particularly important due to the variation caused by the high fat content and correspondingly low cell number per gram of human breast tissue. Tumor tissue contained less fat than normal breast tissue, reflecting a fundamental difference in the cellular composition of the tumor and normal breast. For tumor tissue, but not normal tissue, ascorbate standardised to DNA was significantly correlated to ascorbate per tissue wet weight (Pearson *r* = 0.321, *P* = 0.023). Of 52 tumor samples, 50 provided ascorbate data (96%).

**Fig. 3:**
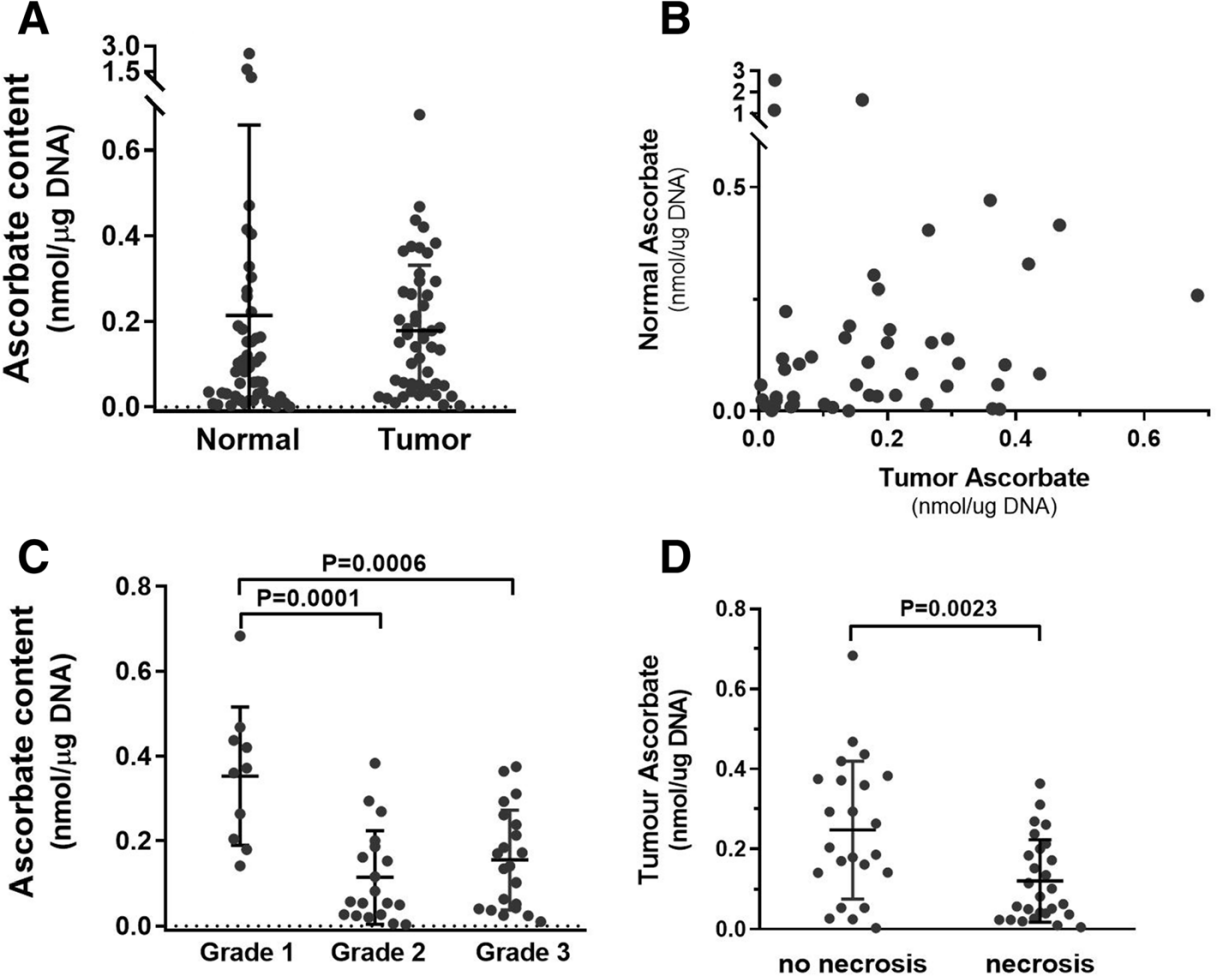
Ascorbate levels in breast tumor tissue and adjacent normal tissue. **a** There was no difference in the range of levels of ascorbate measured in tumor or normal breast tissue samples. **b** There was no association between tumor (*n* = 49) and normal (*n* = 49) breast tissue ascorbate levels. **c** Ascorbate levels were significantly lower in grade 2 (*n* = 20) or 3 (*n* = 22) tumors than in grade 1 (*n* = 9). **d** Ascorbate levels were lower in tumors with necrosis (*n* = 27) than in those without (*n* = 23). Means ± S.D. are shown

Tumor ascorbate levels were similar to those in normal breast tissue (Fig. 3a), but there was no correlation between tumor and normal tissue levels in individual patients (Pearson *r* = − 0.06, *P* = 0.69, Fig. 3b). Both tumor and normal tissue showed a considerable range in ascorbate levels (95% CI 0.136–0.222 and 0.086–0.342 nmol/μg DNA, respectively).

To probe the associations of ascorbate content with patient and tumor characteristics, the cohort was stratified for levels above and below the mean, 0.2 nmol ascorbate/μg DNA (Table 2). Ascorbate levels were generally higher in grade 1 than in grade 2 or 3 tumors (*P* = 0.007, Fig. 3c and Table 2). There was no relationship with tumor stage or tumor size (Table 2), but tumors with DCIS necrosis had lower mean ascorbate content compared with those without necrosis (*P* < 0.01, Fig. 3d). There was no significant difference in the ascorbate levels according to hormone receptor status (Table 2).

**Table 2:** Relationship of ascorbate levels in tumors with clinicopathological and patient parameters

Parameter		Ascorbate in tumor tissue^a^		
		<mean	>mean	*p* value^e^
Ethnicity	Maori/Pacific	7	1	0.099
	non-Maori/non-Pacific	23	18	
	undeclared	1	0	
Age	< 55 year	16	9	0.500
	> 55 years	15	10	
Grade	1	2	8	**0.007**
	2	15	4	
	3	14	7	
Tumor Stage	1	11	5	0.769
	2	19	13	
	3	1	1	
Nottingham^b^	≤ 3.4	4	5	0.370
	3.41–5.4	14	9	
	> 5.4	13	5	
Node	negative	11	5	0.500
	positive	20	14	
Vascular invasion	no	13	10	0.357
	yes	18	8	
	unknown		1	
Tumor size^c^	≤ 2 cm	12	5	0.369
	> 2 cm	19	14	
DCIS Necrosis	no	8	9	0.055
	yes	19	6	
	unknown	4	4	
Hormone Receptor status^d^	HR+	24	17	0.452
	HR-	7	2	

^a^Number of patients below (*n* = 31) or above (*n* = 19) the mean ascorbate level of 0.2 nmol/μg DNA in tumor tissue

^b^Nottingham prognostic index: calculated from tumor size, number of involved lymph nodes and tumor grade [73], with good, moderate and poor prognosis [74].

^c^Tumor size, invasive component only, excluding DCIS

^d^Hormone receptor HR^+^, ER+(PR+/−, Her2+/−) and includes both Luminal A and B; HR^−^, ER-PR-(Her2+/−) and includes Her2-enriched and basal-like tumours

^e^Chi-square or Fisher’s exact test, as appropriate. Significance is indicated when *p* < 0.05

### Association of tumor ascorbate with the HIF pathway

**Fig. 4:**
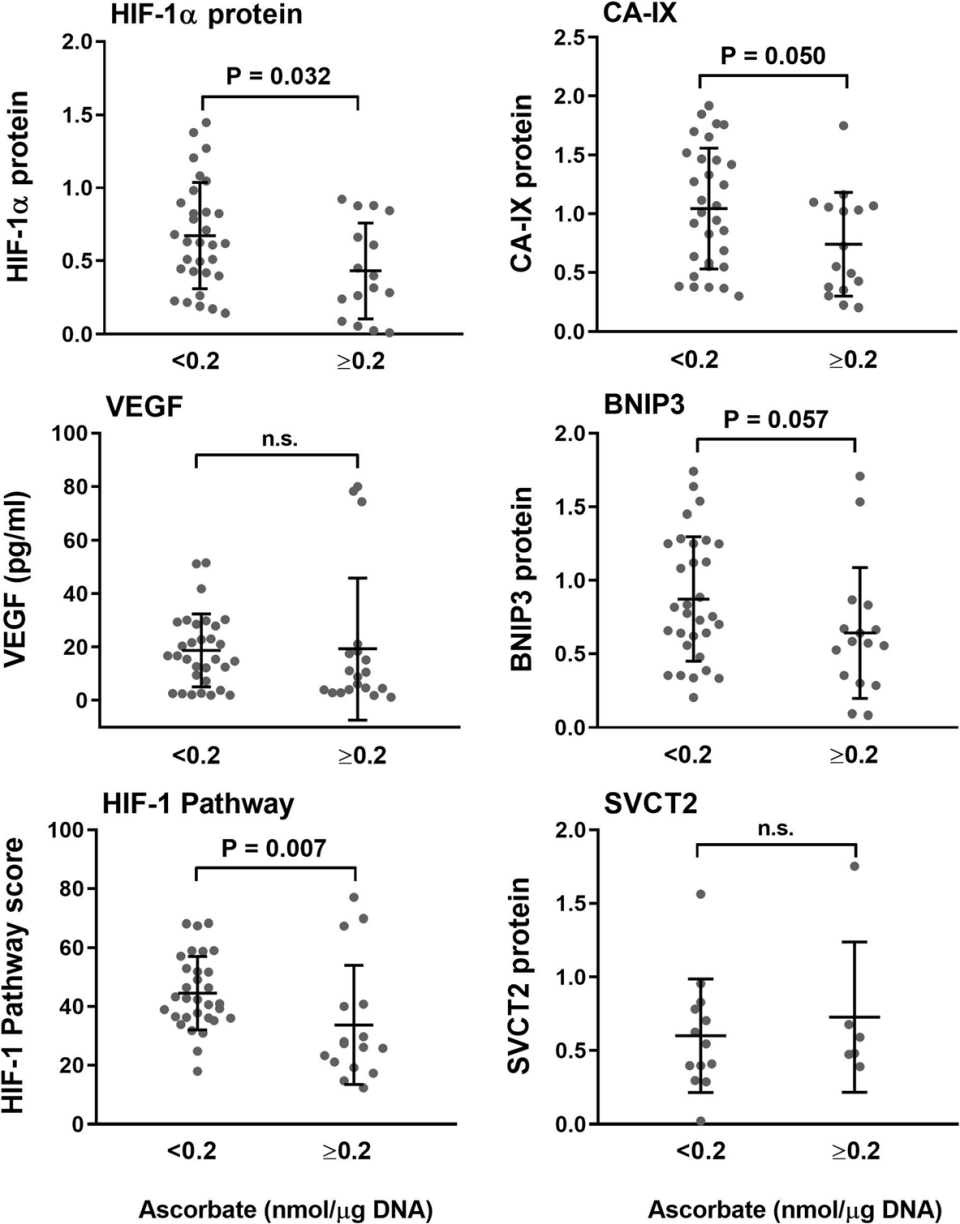
Relationship between tumor ascorbate and expression of the HIF-1 pathway proteins and SVCT2. Levels of HIF-1α, CAIX, VEGF and BNIP3 were stratified according to the mean tumor ascorbate level < 0.2 nmol/μg DNA (*n* = 31), > 0.2 nmol/μg DNA (*n* = 16). HIF-1α, CAIX, VEGF and BNIP3 were generally higher in tumor tissue with low ascorbate, culminating in a significant relationship between higher ascorbate levels and a low HIF Pathway score. Means ± S.D. are shown. Statistical significance was determined with an unpaired t-test (HIF-1α, CA-IX) or the Mann Whitney test for non-parametric data (VEGF, BNIP3, HIF Pathway, SVCT-2). SVCT-2 below the mean *n* = 13, above the mean *n* = 6

The levels of HIF-1α and HIF-1 target proteins were compared between tumors with below or above mean ascorbate levels (Fig. 4). HIF-1α protein and CA-IX levels were significantly higher in tumors containing below mean ascorbate (*P* = 0.032 and 0.05 respectively, Fig. 4). A similar trend was observed for BNIP3 (*P* = 0.057), whereas VEGF showed little association (*P* = 0.217). There was a strong association between tumor ascorbate and overall HIF-1 activation, with a significantly higher HIF Pathway score in those samples with ascorbate levels below the mean (*P* = 0.007). Tumor ascorbate levels were not related to the expression of the ascorbate transporter SVCT2 (Fig. 4).

### Patient survival according to tumor ascorbate and HIF-1 levels

**Fig. 5:**
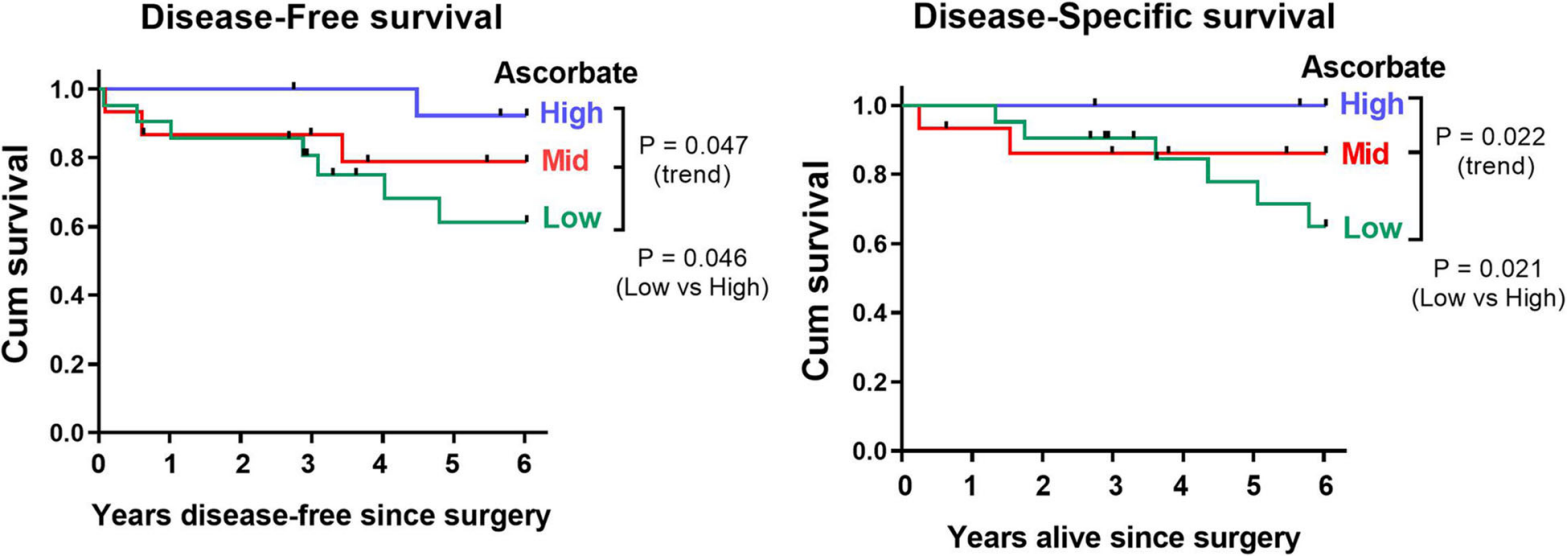
Tumor ascorbate-associated disease-free and disease-specific 6 year survival. Kaplan Meier 6 year survival curves, plotted with respect to tumor ascorbate content (nmol/μg DNA), stratified according to range: Low (< 0.13 nmol/μg DNA, *n* = 21); Mid (> 0.13 - < 0.26 nmol/μg DNA, *n* = 15); High (> 0.26 nmol/μg DNA, *n* = 14). There was a significant association between tumor ascorbate content and both disease-free and disease-specific survival. Significance was determined with the logrank (Mantel-Cox) test and three-way ANOVA for trend with increasing ascorbate levels

There was no significant difference in disease-free survival or disease-specific survival according to mean tumor ascorbate levels (*P* = 0.172 and 0.322, respectively) (Table 3). To determine whether high tumor ascorbate content could contribute to improved survival via the HIF-1 pathway, as suggested by the results in Figs. 2 and 4, and to allow comparison between low and high ascorbate levels, we plotted Kaplan Meier survival curves for the population by ascorbate content, with the tumor ascorbate levels stratified into thirds (< 0.13 nmol/μg DNA; 0.13–0.26 nmol/μg DNA; > 0.26 nmol/μg DNA). As shown in Fig. 5, patient disease-free survival (*P* = 0.047) and disease-specific survival (*P* = 0.022) improved significantly with increasing tumor ascorbate levels. The difference was noted across the range, and also for those patients with the highest tumor ascorbate levels compared with the lowest group (*P* = 0.046 and 0.021 for disease-free and disease-specific survival, respectively).

**Table 3:** Survival of breast cancer patients according to mean tumor ascorbate or HIF-1 pathway score

		Tumor ascorbate^a^		HIF-1 pathway score^a^	
		<mean	≥ mean	<mean	≥ mean
Disease-free survival	Mean days	2772	3613	3865	2412
	95% CI	(2142, 3402)	(3022, 4204)	(3286, 4443)	(1787, 3037)
	No. events	10	4	3	11
	*p* value	0.173		**0.01**	
Disease-specific survival	Mean days	2977	3613	4216	2439
	95% CI	(2350, 3603)	(3022, 4204)	(3834, 4598)	(1824, 3054)
	No. events	8	4	1	11
	*p* value	0.322		**0.001**	

^a^Tumor ascorbate mean: 0.2 nmol/μg DNA, *n* = 31 below the mean, *n* = 19 above the mean; Tumor HIF-1 pathway score median: 41.8 (HIF-1 pathway score calculated from relative protein levels of HIF-1α, CA-IX, BNIP-3 and VEGF); *n* = 28 below and *n* = 21 above the mean. Significance is indicated when *p* < 0.05

## Discussion

Our retrospective analysis of human breast cancer tissue demonstrates a close association between activation of the HIF-1 transcription factor and patient survival. There was a greater difference in disease progression and breast cancer survival when the HIF Pathway score was used than when HIF-1α protein was considered, supporting the use of the pathway score as a robust marker of HIF-1 transcriptional activity and, potentially, patient prognosis. Most other studies that have monitored hypoxia markers used a single protein marker of HIF-1 activation [5–9, 11–16]. We found that the tumor grade, stage, size, vascular invasion, and necrosis were also associated with a high HIF Pathway score, but not with HIF-1α alone. This data supports the use of a HIF-1 signature that combines measures of HIF-1α and downstream genes and highlights the usefulness of the HIF Pathway when monitoring the impact of transcriptional activity. Similarly, a HIF-1 expression signature of 16 genes was shown to be more tightly associated with patient survival from breast cancer than was the expression of HIF-1α alone [48]. Together, these observations reflect the prevalence of HIF-1 transcriptional activity in breast cancer and underscore the clinical interest in modulating this response to improve chemotherapy response and decrease metastasis.

There is some evidence that breast cancer sub-types may be differentially affected by HIF-1. One study has shown that HIF-1 preferentially stimulates cancer stem differentiation in ER+ but not ER- breast cancer cells [49]. We also noted significant difference in HIF-1α protein and HIF-1 pathway score between hormone receptor positive and negative tumors. The HR+ group includes both Luminal A and B sub-types, whereas the HR- group includes Her2-enriched and basal-like tumours [50]. However, we saw no difference in ascorbate levels according to HR status.

Elevated tumor ascorbate was associated with decreased expression of HIF-1α protein and downstream genes, and was strongly associated with a low HIF Pathway score, suggestive of an overall effect of ascorbate on HIF-1 activation. It is highly likely that this association reflects activity of ascorbate as a co-factor for the HIF hydroxylases. Although we are unable to test this directly in our retrospective tumor tissue analysis, this effect has been documented in in vitro studies where low intracellular ascorbate levels exacerbate HIF-1 activation in response to moderate hypoxia [25, 30] by affecting the proline and aspargine hydroxylases [25, 51]. Our recent analysis of renal cell carcinomas also provides support for a role for ascorbate as a modulator of HIF-hydroxylase activity. We found no association between ascorbate levels and HIF pathway activation in clear cell renal cell carcinoma, which lacks a functional von Hippel-Lindau (VHL) protein, resulting in constitutive activation of HIF [33]. This contrasted with a clear association between ascorbate content and the HIF pathway observed in papillary renal cell carcinomas with normal VHL and a functional hypoxic response [33].

In addition to ascorbate, low O_2_ supply and availability of metabolic intermediates will influence HIF-1 activity [1–3]. This is apparent in our results, as a low HIF Pathway score was not confined to samples with high ascorbate levels. This is in agreement with our knowledge that the HIF hydroxylases are inhibited by lack of O_2_, 2-oxoglutarate or iron [51–53] resulting in the up-regulation of HIF-1. Optimal intracellular availability of oxygen, 2-oxoglutarate and iron, in supporting HIF hydroxylase activity, should help limit HIF-1 activation. Our observation that those tumors with ascorbate levels above the mean had the lowest HIF Pathway scores indicates that increased levels of intracellular ascorbate help optimise HIF-1 hydroxylase activity and could mitigate against HIF-1 activation.

Our study is the first to measure both tumor ascorbate levels and the HIF Pathway in human breast cancer and to demonstrate the potential for moderation of HIF by ascorbate in this disease. Given the importance of HIF-1 as a driver of breast cancer progression [5–9, 11–16], our findings could be of clinical significance and the stratified analysis of disease-free and disease-specific survival underscores this potential.

The tumor ascorbate level varied across a significant range, from 0.002–0.683 nmol/μg DNA. The median was relatively low on this scale (0.15 nmol/μg DNA), which suggests that ascorbate levels may be compromised in many tumors. Ascorbate is delivered via the vasculature, and when plasma levels are low or when the tissue is poorly perfused, cellular uptake is likely to be limited. Our modelling of ascorbate transport through the tissues has predicted a similar distribution profile for ascorbate as for oxygen [54], reinforcing the potential for a double impact of both oxygen and ascorbate deprivation on the HIF-1 activation. Ascorbate levels in breast tumors were not generally related to normal breast tissue levels, and we found no association between tumor ascorbate levels and expression of SVCT-2, the major ascorbate transporter [55–58]. These findings suggest that numerous factors could affect ascorbate delivery in tumors, with a combined impact of SVCT-2 protein expression and variable plasma supply. SVCT-2 levels were shown to correlate with ascorbate uptake and to influence chemo-sensitivity in breast cancer cells in vitro [59]. Low plasma ascorbate levels have been documented in breast cancer patients, with levels as low as 15 μM [60] or 36 μM being reported [61–64]. In contrast, plasma levels in the general population average 50 μM, with levels above 70 μM being indicative of plasma saturation [65–68]. The broad range of ascorbate levels in our cohort of breast cancer tissue suggests a complex interplay between tumor vascularity and perfusion, plasma ascorbate availability and variable SVCT-2 expression.

Some recent studies have indicated that plasma levels in excess of 100 μM ascorbate are required to overcome the perfusion barrier in tumors [54, 69–72]. Whether this is the case and how best to effectively raise tumor ascorbate levels in order to down-regulate the HIF Pathway are matters for future investigation. Our study suggests that tumor ascorbate levels are an important consideration in the effort to mitigate against the detrimental clinical outcome when HIF-1 transcriptional activity is elevated.

## Conclusions

Our retrospective analysis of tumor tissue has determined that tumor ascorbate content is associated with decreased activation of the HIF-1 pathway in human breast cancer. Low levels of HIF-1 activation were strongly indicative of a favourable prognosis and the data suggest that elevated tumor ascorbate levels could moderate the hypoxic response in breast tumors, with a potential clinical benefit. This hypothesis should be tested in future clinical intervention studies.

## Abbreviations

BNIP-3: Bcl-2/adenovirus E1B 19 kDa interacting protein 3
CA-IX: Carbonic-anhydrase IX
CSTB: Cancer Society Tissue Bank
DCIS: Ductal carcinoma in situ
DFS: Disease-free survival
DSS: Disease-specific survival
ER: Oestrogen receptor
Her2: Human epidermal growth factor receptor 2
HIF-1: Hypoxia-inducible factor-1
HR: Hormone receptor
IDC: Invasive ductal carcinoma
PR: Progesterone receptor
SVCT-2: Sodium-dependent vitamin C transporter - 2
VEGF: Vascular endothelial growth factor
VHL: Von Hippel Lindau
